# Medical Opinion and Movement

**Published:** 1909-05-01

**Authors:** 


### Medical Opinion and Movement.

THE Experimental Production of Choked Disk has been the object of investigation by Drs. H. Cushing and J. Bordley, Jun., in the Hunterian Laboratory of the Johns Hopkins Hospital. They find that the introduction of fluid under tension into the intra-cranial subdural space will produce an acute œdematous swelling of nerve head and retina—that is, a choked disk—which can be detected both by the ophthalmoscope and by post-mortem examination. Simple digital compression through a trephine wound produces the same result; and distension of the optic sheath accompanies both this and the fluid pressure. On the other hand, venous congestion alone cannot produce more than the congestive features of optic neuritis. With the idea of simulating the increasing pressure of a growing tumour they then introduced fragments of compressed sponge tent between the skull and the dura. These swelled gradually, and caused all the signs of choked disk as it is seen in spontaneous tumours: whereas a solid aseptic body which does not alter in size does not cause such changes. They pronounce, therefore, strongly in favour of the strictly mechanical theory of the production of optic neuritis, and express the view that decompression by tapping the cerebro-spinal fluid may be followed by subsidence of the process, even when it is of considerable extent: provided, that is, that the stage of new tissue formation has not been reached.

A CURIOUS inter-relation of the Pancreas and the Supra-renal Bodies functionally seems to be revealed by certain experiments and deductions of Loewi, published in the *Archiv. für Experim. Path. und Phar.* In health suprarenal extract does not cause any dilatation of the pupil, although its action in the main is one of stimulation of sympathetic nerve fibres. This holds good both of man and of the lower animals, such as cats and dogs. But in the two latter after artificial total extirpation of the pancreas, suprarenal extract does dilate the pupil; and Loewi has further established that the same occurs in man in some cases of pancreatic insufficiency and in many cases of diabetes considered to be of pancreatic origin. The same is true of many cases of exophthalmic goitre. He concludes from these observations that the pancreas may have a function of inhibiting the action of the suprarenal glands upon parts of the sympathetic nervous system, and that the pupillary reaction he describes may assist in the diagnosis of diabetes and of pancreatic lesions in the human subject.

A NEW method of Recognising and Localising Ulcers of the Stomach and Duodenum is described in the *Medical Record* by Dr. Max Einhorn. He calls his proceeding the "thread impregnation test," and carries it out thus. At 9 P.M. the patient who is suspected of such an ulcer swallows a duodenal bucket in a gelatine capsule. The string is of braided silk (No. 5, English), and is moored to the shirt in such a way that the bucket can pass downwards for 75 cm. (about 30 inches) from the lips. The apparatus is removed at 7 or 8 A.M. the next morning, the patient fasting until then. In favourable cases, particularly if the thread has come into intimate contact with the surface of the ulcer, a brown or dirty black discolouration reveals the distance of the lesion down the digestive tract. At 40 cm. from the lips an ulcer of the cardiac end of the stomach is indicated; at 44 to 54 cm. of the lesser curvature; at 56 to 58 cm. of the pylorus; and at 59 and upwards of the duodenum. Œsophageal ulcers can also be diagnosed by the same means; but those of the fundus and greater curvature are usually not disclosed. Dr. Einhorn has also found the method useful in judging the result of treatment: after liquid diet it is less marked than after coarser food. Simple ulcers cannot be differentiated from malignant. In order to locate ulcers of the fundus and greater curvature the author is experimenting with what he calls a "gastric stamper." This is an inflatable rubber balloon covered with silk gauze, which, when blown up through a tube after being swallowed, takes the shape of the stomach, and registers on the gauze a blood-stain from any ulcerated part. After half an hour, during which time the patient remains recumbent, the air is allowed to escape from the balloon, which is then withdrawn and blown up again. This latter technique can be done only upon those accustomed to lavage: the silk impregnation test can be carried on on anyone.

AT the recent German Medical Congress held at Wiesbaden, Dr. F. Widal contributed an interesting paper to the discussion on the Dechlorination Treatment of Bright's Disease. It will be remembered that this method of treatment owes its origin to a large extent to the researches of Dr. Widal. In collaboration with others he showed that the œdema of Bright's disease is the result of an accumulation in the tissues of the excess of sodium chloride ingested, which the kidneys, in their morbid condition, are unable to eliminate. By controlling the proportion of sodium chloride in the diet he has been able to cause œdema to disappear and reappear in patients afflicted with Bright's disease. The usual milk diet prescribed for these patients is found to be less suitable than an ordinary mixed diet deprived of salt. Reckoning the milk diet at 3 litres a day, it contains four times as much salt as an equivalent mixed diet deprived of salt, and includes nearly 3 litres of water and 120 grammes of albumen, which are considered altogether too much for many of those patients. The amount of chlorides that can be borne by a patient varies in different cases, and, after excluding it entirely from the diet until the œdema has disappeared, it is often possible to make certain additions without giving rise to recurrence of the symptoms. In the case of a milk diet about 1.6 gm. of salt are absorbed per litre. With a mixed diet without any addition of salt about 1.5 gm. of salt are taken per day, but with a specially restricted diet this may be reduced to 1 gm. per day. A dechlorinated diet not only arrests the œdema, but also affects the disease itself.

THE author distinguishes two conditions in the disease—chloræmia, in which there is retention of chlorides in the system; and uræmia, with retention of nitrogen products. When the kidneys fail to eliminate the chlorides from the system they are retained in the tissues, and by a process of osmosis they attract the fluid into the tissues also and give rise to œdema. On the other hand, the excess of urea accumulates in the blood, and the author is convinced that the œdema is in no way attributable to this agent. In many cases, especially in the last stages, the two conditions may be combined. In view of these facts the author emphasises the importance of ascertaining the proportion of urea circulating in the blood, as the presence of uræmia seriously affects the prognosis and the results that may be expected from the dechlorination diet. In cases of considerable chloride retention and marked œdema the urea may not exceed 20 to 50 centigrammes per litre of blood, and in such cases the chloræmia is the chief factor. When, however, the proportion of urea exceeds 1 gramme per litre, the presence of uræmia must be assumed, and the same expectation of a cure by the dechlorination diet cannot be entertained. In the discussion which followed several physicians testified to the excellent results obtained by the dechlorination treatment in cases of Bright's disease, and expressed the conviction that the symptoms of anasarca are due to the retention of chlorides in the tissues. Dr. Magnus-Levy pointed out that although the anasarca due to cardiac disease and cirrhosis of the liver is of a mechanical nature and cannot be affected in the same way, he found that in these conditions also a dechlorinated diet has the effect of reducing the dropsy.

---

AT the recent congress of the German Surgical Society at Berlin, there was the usual discussion on Anæsthetics. Neuber, of Kiel, gave some interesting statistics in favour of the use of scopolamine injections previous to operations. As a result of his inquiry into the administration of anæsthetics during the past year he finds that out of more than 71,000 administrations, chloroform was used in 20,615 cases, ether in 11,859 cases, a mixture of ether and chloroform in 10,232, and Billroth's mixture in 2,791. This shows a marked diminution in the use of chloroform. On the other hand scopolamine appears to have gained considerably in favour, as it is mentioned in 23,809 cases. The mortality figures are one death in 2,060 cases for chloroform, one in 5,930 for ether, one in 3,410 for the mixture of chloroform and ether, one in 698 for Billroth's mixture, and one in 4,762 for scopolamine. The author is of opinion that the mortality from ether has been largely reduced owing to the more general adoption of the open method of administration. He is strongly in favour of the preliminary injection of scopolamine-morphine. He injects 0. gr. 0005 decimilligr. of scopolamine and 0. gr. 01 centgr. of morphine an hour before operation. The advantages claimed are that the anæsthesia need not be deep, and it is unnecessary for the reflexes to disappear. There is no previous anxiety on the part of the patient, and in consequence of the light degree of anæsthesia complications are avoided. Out of 300 consecutive anæsthesias there were no complications except one case of pneumonia. Other members also spoke in favour of scopolamine injections, but Sprengel, of Brunswick, stated that he had abandoned the use of the drug in consequence of a considerable increase in cases of pneumonia.

---

THE Surgical Treatment of General Suppurative Peritonitis formed another subject for discussion. Nearly all those who took part in the debate were in favour of thorough lavage with normal saline and drainage by means of tubes. On the other hand Sprengel and Roth adhered to the opinion that general lavage is dangerous and should be avoided, as it is liable to spread the infection and break down the natural barriers of resistance of the organism. There was also a good deal of difference of opinion shown in regard to the closing of the abdominal wound. While many prefer to close the wound entirely, except for the small openings left for the drainage-tubes, others advocate only a partial closure. Bochard, of Posen, injects sterilised olive oil into the peritoneal cavity on the ground that it hinders absorption by the peritoneum. He thinks that it also covers the irritated surfaces and prevents the formation of adhesions and intestinal paralysis. He injects 50 to 100 cubic centimetres. He uses lavage only in a moderate degree and does not drain. He sutures the peritoneum and muscular layers, but not the skin. By this method of technique he claims to have a mortality of only 26 per cent.

---

PROFESSOR PONCET and Dr. Lericke describe in *International Clinics* Articular Manifestations of Tuberculosis which are, they believe, frequently mistaken for ordinary acute rheumatism. The clinical signs of this Acute Tubercular Rheumatism differ very little from those which are usually held to be diagnostic of the real thing: the onset is sudden, the large joints are chiefly selected, the temperature and pulse-rates are both much raised. Further, there may be recurrences separated by intervals of complete health. The inflammation may flit from joint to joint; it may subside altogether in a few days, or assume a sluggish relapsing character with attacks of pain, or settle tenaciously in one joint which is ultimately crippled. Sometimes there are simultaneous inflammations of the pleura, pericardium, etc.; sometimes not. Salicylate treatment has no influence over these affections, which may be gradually succeeded by definite pulmonary or other tubercular symptoms, or may precede by years the complaints which finally, according to these authors, establish the true diagnosis of the articular trouble. In one of the numerous types described, the most affected joint becomes ultimately the seat of local tuberculosis. Consecutive or secondary acute articular rheumatism is also described as not uncommon in those already the subject of gross tuberculosis in other regions. The authors catalogue so many clear types of tubercular rheumatism that it seems very strange that such definite symptom complexes should hitherto have passed unrecognised.

THE *Sleeping Sickness Bulletin* for March, edited by Dr. A. G. Bagshawe, contains some very interesting papers on the Development of Trypanosomes in tsetse flies and the mode of transmission of trypanosomes by tsetse flies. Recently it has been a point of dispute as to whether the trypanosomes when sucked up by the tsetse fly develop in it, and so far most of the work done has seemed to show they do not. In the present edition of the bulletin, however, Dr. Bagshawe quotes a very important paper by Professor Kleine, from German East Africa, which, if confirmed, would seem to prove that they do. According to Professor Kleine, flies which for many days after the ingestion of blood containing trypanosomes were not infective afterwards became so, and infected a sheep and then an ox. This would place the subject on the same footing as malaria in the mosquito. It is only after 12 days or so, during which time the malarial parasite has been developing, that the mosquito becomes infectious. In Professor Kleine's series of experiments with trypanosomes in tsetse flies nothing happened where animals were bitten by infected flies up to the 17th day or longer, but after that infection is said to have taken place. The whole subject is of so great importance that it is to be hoped that these experiments will be quickly confirmed or refuted by other observers.

---

A MOST important research upon the Ætiology of Endemic Goitre has been carried out in Kashmir by Captain R. McCarrison, I.M.S. As a result of experiments upon himself and several other people who volunteered to be made the subjects of research, he has shown that goitre can be set up by the administration of the suspended matter removed by filtration from waters which are known to be goitre-producing. When this suspended matter is boiled before being ingested no goitre is produced: the disease is therefore due not to mineral, but to biological components of the suspended matter; in other words, to a pathogenic micro-organism. The incubation period of goitre thus experimentally produced is thirteen to fifteen days, and it can be cured by the administration of intestinal antiseptics. It seems probable, therefore, that the specific organism is parasitic in the human intestine: in the fæces of most of those who suffer from goitre in Kashmir an amœba is plentifully found, but whether this has anything to do with the disease is not yet established. In view of these remarkable findings it would be of interest to know whether the same facts hold good of endemic goitre in other parts of the world, especially Switzerland.

---

TO the tuberculin ophthalmo- and cuti-reactions must now apparently be added a third—Mantoux's Intra-dermo Reaction—recently described in *La Presse Médicale.* One c.c. of a 1-5000 tuberculin solution (Pasteur Institute) is injected in the manner indicated. Positive signs are infiltration in a few hours, and after twenty-four a surrounding area of erythema; in forty-eight the reaction is at its height. General disturbance usually fails, although there is sometimes some fever. The reaction is more acute than Pirquet's cuti-reaction, and it is also stated that whereas the latter gave nine negative results out of 52 children tried, these nine cases came positive with the intra-dermo reaction. Professor Hutinel reports on it after a more extended trial that it is the most certain and safest of the three tests, with the qualification that it only indicates that the organism is tuberculous, not that any given lesion is so. He recommends it especially for children, although one would think that the fact that it must often be a rather painful procedure would be some slight hindrance to its use in pædiatrics. Two other recent papers are of interest in the above connection. Chauffard and Troisier in the *Bulletin Médicale* say that in the course of their trial of the intra-dermo reaction they were struck by the resemblance of the appearances obtained to those of erythema nodosum, having been able to produce at will in a tuberculous subject typical nodules of this affection. They put the question whether erythema should be considered a manifestation of bacillary septicæmia—in short, an atypical cutaneous tuberculide. Lévy-Franckel (*Revue de la Tuberculose*, No. 5) tried the ophthalmo-reaction on three subjects of erythema nodosum, with a positive result in each case. In spite of this, however, he does not believe in a tuberculous nature of this disease.

---

THE position of graduate students of medicine, and, indeed, the whole subject of Graduate Study in Medicine will be discussed by a special committee at the forthcoming International Congress of Medicine to be held at Buda Pest during the present summer. Professor Kuttner, the veteran leader of the graduate study movement in Germany, who is so well known to all those who take an interest in the movement, for the good work that he is doing at the Kaiserin Friedrich Haus in Berlin, will adequately represent the German graduate study societies and associations, while other representatives will be sent from Belgium, France, Holland, Russia, Italy, and America. It is to be hoped that England will be suitably represented, and that the labours of the committee, which, as we understand, will be mainly directed to the creation of an international committee, will be crowned with success. The committee which it is contemplated to start will be a permanent one, with representatives in all large cities where there exist facilities for graduate study. It will cater for the graduate student exclusively, and will attempt to do for him in international matters what the present local committees are doing in provincial matters. There is, we believe, urgent need for such a committee, and its creation will be cordially welcomed by the large number of present-day graduates, since it will enable them to obtain first-hand and reliable information concerning the special points on which they desire to be enlightened with regard especially to time, place of study, classes and fees. The committee will work in unison with the established medical Press in the various countries, and will from time to time publish official notices and pamphlets for the benefit of graduate students.

